# Cross-lagged analysis of problematic social media use and phubbing among college students

**DOI:** 10.1186/s40359-023-01062-0

**Published:** 2023-02-10

**Authors:** Shuai Lv, Hui Wang

**Affiliations:** 1 School of Modern Logistics, Qingdao Harbour Vocational and Technical College, Qingdao, People’s Republic of China; 2 Department of Physical Education, Xiamen University Tan Kah Kee College, Zhangzhou, People’s Republic of China

**Keywords:** College students, Problematic social media use, Phubbing, Cross-lagged analysis, Reciprocal relationship

## Abstract

**Background:**

Phubbing is a commonly seen phenomenon that has emerged in recent years among groups of college students, posing a rising challenge to educators. We conduct research in which the reciprocal relationship between problematic social media use and phubbing is explored and analysed quantitatively, aiming to discover reliable theoretical support to work out an appropriate intervention on students’ phubbing for students’ mental health concerns.

**Methods:**

Using the problematic social media use scale and the phubbing scale, 328 college students from four universities in Shandong Province were enrolled in a two-stage longitudinal follow-up study for 20 months from December 2019 to August 2021. A cross-lagged model was constructed to explore the reciprocal relationship between problematic social media use and phubbing. The results of correlation analysis showed that problematic social media use was positively correlated with phubbing at both time points (r = 0.51, 0.53, *P* < 0.01).

**Results:**

The results of cross-lagged regression analysis showed that the predictive effect of pretest problematic social media use on posttest problematic social media use was statistically significant (β = 0.24, *P* < 0.01). There was statistical significance in the prediction effect of pretest phubbing on posttest phubbing (β = 0.16, *P* < 0.05). Pretest problematic social media use had statistical significance in predicting posttest phubbing (β = 0.22, *P* < 0.01), and there was no statistical significance in the prediction of pretest phubbing on posttest problematic social media (β = 0.16, *P* < 0.05).

**Conclusion:**

The problematic social media use of college students is closely related to phubbing, and problematic social media use can predict phubbing.

## Background

In the information age, especially with the rapid development of the internet, the prevalent usage of smartphones has changed the manner of daily communication and interaction within the college student community, which can both enhance long-distance social connections and destroy off-line face-to-face interaction [1, 2]. Phubbing is a behaviour that has arisen in conjunction with the widespread use of smartphones [3, 4], in which individuals play on their smartphones and ignore the surrounding people or things in social situations [5]. Studies have shown that phubbing not only affects impression formation and communication quality between communicating parties and impairs the quality of peer relationships between college students [6–8] but also reduces the well-being of interpersonal objects [9, 10]; there are even studies that have found that phubbing among college students affects their performance in class [11, 12], leading to poorer academic performance [13, 14] and even cheating behaviour [15]. Phubbing has already been found to have a negative impact on the physical and mental development of college students. Thus, it is important to explore the causative factors of phubbing among college students to stimulate their physical and mental health into better development.

Phubbing is influenced by several factors, of which problematic social media use is one of the most important. Problematic social media use is defined as “unhealthy excessive social media use behaviours” [16]. Problematic social media use has emerged as a maladaptive pattern of social media use; it has been characterized by excessive concern about social media, being driven by a strong motivation to use social media, and devoting so much time and effort to social media use that it impairs other social activities, studies, jobs, interpersonal relationships, psychological health and well-being [17]. Numerous studies have indicated that problematic social media use is associated with a range of negative consequences; furthermore, when problematic social media use is restricted, adolescents experience symptoms of physical discomfort such as stress or anxiety and are constantly presented with scenarios of problematic social media use in their minds [18–20]. In addition, problematic social media use may also reduce adolescents' well-being at home and school [16, 21]. Therefore, problematic social media use is a phenomenon to which researchers have paid focused attention. Recently, several studies have linked this phenomenon to phubbing to discuss the potential relationship and influential mechanism between the two [9, 22–25].

There are two main perspectives on the relationship between problematic social media use and phubbing. The first perspective suggests that problematic social media use can influence phubbing. According to the limited capacity model view [26], individuals have limited cognitive or mental resources to process information. If individuals are exposed to large amounts of information due to the use of online platforms and technological devices, their mental resources become strained and can become overloaded. We expect that when individuals experience problematic social media use, they will be overloaded with mental strain, have no time for their partners, and be cold to others in reality because of their heavy exposure to online information. According to self-determination theory [27], relatedness (i.e., closeness or connectedness with others) is one of the three basic psychological needs of human beings and is critical to achieving the optimal development of the individual. The need for relatedness will induce college students’ desire to seek emotional support from social media, especially when the needs in the offline context are not well satisfied. The more emotional support is provided by social media, the less likely college students are to seek face-to-face emotional support and the more likely they are to phub their offline interaction peers. The displacement hypothesis also proposes that an increase in overall time on social media makes less time available for more beneficial face-to-face emotional support [28]. Thus, problematic social media use may cause phubbing in college students. In addition, researchers have claimed that phubbing is considered a new form of addiction and that as the availability of virtual environments created by online social media continues to increase, a number of problematic mobile technology use factors have been identified as valid predictors of phubbing, with individuals who do not use their smartphones appropriately being more likely to be apathetic in social situations [22]. Similarly, it has been shown that individuals who overuse social media are also more likely to use their smartphones in social situations, thereby leaving others aside [23, 24]. Thus, problematic social media use is one of the main reasons for phubbing.

The second perspective suggests that phubbing can influence problematic social media use. While the stated purpose of technology such as smartphones is to help us connect with others, in some particular cases, technology that is designed to bring humans closer together isolates us from those people. Researchers argue that individuals with high degrees of phubbing experience a sense of social exclusion that leads to a high need for attention and that this need for attention in turn leads individuals to attach to social media in the hope of regaining a sense of inclusion, ultimately causing problematic social media use [9]. At the same time, similar studies have found that phubbing disrupts real-life interpersonal relationships, leading individuals to turn to online social media to satisfy the need for interaction [25], resulting in dependence on social media and problematic social media use. Thus, phubbing is one of the main causes of problematic social media use.

In summary, we aimed to explore the relations between problematic social media use and phubbing over time. Our study enriches this field in two ways. First, most of the above studies exploring the relationship between phubbing and problematic social media use are based on the results of cross-sectional design studies, which are still essentially correlations and cannot truly and strongly reflect the reciprocal relationship between the two. To investigate the reciprocal relationship between phubbing and problematic social media use, this study adopts a longitudinal design. Second, the university period is a critical developmental stage in life, where the psychology and behaviour of individuals undergo certain changes, especially in today's digital age, and where college students may change beyond our expectations. To explore the changing trends of problematic social media and phubbing among college students, we conducted a longitudinal survey of Chinese college students. This study conducted a longitudinal tracking survey at two different designated times, with an interval of 20 months between December 2019 (T1, below) and August 2021 (T2, below), to construct a cross-lagged model to analyse the reciprocal relationship between problematic social media use and phubbing among college students, with a view to providing a follow-up study. A cross-lagged model was constructed to analyse the reciprocal relationship between problematic social media use and phubbing among college students to provide a reference for subsequent studies.

## Methods

### Participants

Using a whole-group sampling method, 10 freshman classes in 2019 from four universities in Shandong Province, China, with a total of 380 college students, were selected as subjects. The first measurement was conducted in December 2019 when the subjects were in their first year of college, and 380 questionnaires were distributed; the second measurement was conducted in August 2021 at the end of the subjects' second year of college for the same group of students, and 364 questionnaires were distributed (16 of the subjects were lost due to suspension and withdrawal). The students' school ID number information was used for matching, and due to some students' misfiled school ID numbers and personal reasons for refusing to fill in their real school ID numbers again, 328 valid paired questionnaires were finally matched for the pre- and postmeasurement data. Among them, 136 were male and 192 were female; 180 were urban students and 148 were rural students. On average, the age of the participants was 18.82 years (SD = 0.80) at Time 1. The survey was conducted with the informed consent of the subjects and approved by the Ethics Committee of Qingdao Harbour Institute of Technology.

### Problematic social media use scale (PSMUS)

The scale was adapted from the Compulsive Internet Use Scale (CIUS) by Franchina et al. [29] and contains 7 items, with the sample question "I find it difficult not to use social media". The scale is scored on a scale ranging from 1 (strongly disagree) to 5 (strongly agree), and all the statements are positive; the higher the total score is, the less healthy the individual's use of social media is. In the current study, the scale had Cronbach alpha coefficients of 0.80 and 0.83 at 2 time points.

### Phubbing scale (PS)

The scale was developed by Qiu [30] and consists of 8 items, such as "I spend a lot of time on my smartphone when I am with my friends" and "Sometimes I do not even notice when my friends leave because I am staring at my smartphone". The questions are scored on a scale ranging from 1 (strongly disagree) to 5 (strongly agree), and all the statements are positive; the higher the total score is, the higher the individual's level of phubbing is. In this study, the scale had Cronbach alpha coefficients of 0.89 and 0.94 at 2 time points.

### Statistical analyses

First, Harman’s single-factor method was applied to test the data for common method bias. Second, the variables were tested for descriptive statistics (means and standard deviations), Cronbach's alpha coefficients, and correlations at Time 1 and Time 2. Third, the measurement invariance of the measurement model across time was assessed. Three steps, namely, configural invariance, weak invariance and strong invariance, were included. The most important of all is the inclusion of configural invariance, which sets the baseline model and tests whether the morphological composition of the latent variables is the same. In addition, weak invariance tests the equivalence of factor loadings across groups between the measures and factors on the basis of satisfying the baseline model; finally, strong invariance limits the weak invariance of the observed variables on the basis of satisfying the unit equivalence. Fourth, to explore a possible causal relationship, Martens and Haase suggested testing four‐nested models; in the current study, M1 is a baseline model that only includes autoregression and tests the lateral stability of problematic social media use and phubbing between T1 and T2 [31]. M2 is based on M1, with the addition of problematic social media use from the previous time point downwards to phubbing at one point in time. M3 builds on M1 by adding a similar but opposite path as the cross-lagged path used in M2, that is, from phubbing at one point in time to problematic social media use at the next point in time. M4 is the full model containing all the paths described in the above mentioned models. By comparing the four models above, a chi-square test was used to determine the nested model that provided the best fit, that is, a likely causal relationship.

## Results

Since both the Questionable Social Media Use Scale and the Phubbing Scale were answered by the same group of college students, some common method bias may exist. In this study, common method bias was tested using Harman's one-way test, and exploratory factor analysis was conducted on all variables. The results showed that the first factor with a characteristic root greater than 1 explained 30.02% of the variance, which was less than the critical value of 40 [32]. Therefore, there was no significant common method bias present in this study.

The results of the descriptive statistical analysis and paired-samples t test for the two variables of problematic social media use and phubbing among college students at T1 and T2 are shown in Table 1. As shown in Table 1, although the scores of problematic social media use among college students increased from T1 to T2, the difference between them was not statistically significant (t = − 1.49, *P* > 0.05), while the scores of phubbing significantly decreased (t = 3.95, *P* < 0.05).

**Table 1:** Descriptive results of problematic social media use and phubbing

	T1		T2		t value	*P* value
	M	SD	M	SD		
Problematic social media use	2.47	0.68	2.53	0.69	− 1.49	0.14
Phubbing	1.99	0.68	1.81	0.70	3.95	0.00

T1 represents the first measurement, and T2 represents the second measurement

The results of the correlation analysis between problematic social media use and phubbing among college students as measured at both the T1 and T2 time points are shown in Table 2. In terms of simultaneous correlation, there was a significant positive correlation between problematic social media use and phubbing at both the T1 and T2 time points (r = 0.51 and 0.53, *P* < 0.01). In terms of ephemeral correlations, there was a significant positive correlation between T1 problematic social media use and T2 phubbing (r = 0.27, *P* < 0.01), and T1 phubbing was significantly positively correlated with T2 problematic social media use (r = 0.22, *P* < 0.01). In summary, the above results are consistent with the prerequisite hypothesis for conducting cross-lagged analysis.

**Table 2:** Correlation between problematic social media use and phubbing

Variable	1	2	3	4
T1 problematic social media	–			
T1 phubbing	0.51**	–		
T2 problematic social media	0.25**	0.22**	–	
T2 phubbing	0.27**	0.28**	0.53**	–

T1 represents the first measurement, and T2 represents the second measurement. *P < 0.05;**P < 0.01;***P < 0.001

Before conducting the longitudinal modelling, we tested the measurement invariance for the Time 1 to Time 2 constructs to make inferences about changes in constructs over time [33]. Measurement invariance was established if the CFI did not change more than 0.02 and the RMSEA did not change more than 0.03 for the invariant model [34].

As shown in Table 3, the weak invariant model with equal factor loadings did not show a meaningful deterioration in model fit (for week invariance, ΔCFI = 0.020, ΔRMSEA = 0.006). Taken together, the results support the longitudinal measurement invariance of the constructs across time.

**Table 3:** Statistics of measurement invariant models

Model	χ^2^	df	CFI	SRMR	RMSEA	ΔCFI	ΔRMSEA
Configural invariance	841.198	384.000	0.912	0.049	0.060		
Week invariance	959.062	397.000	0.892	0.068	0.066	0.020	0.006
Partial strong invariance	1047.143	407.000	0.877	0.069	0.069	0.015	0.003

The strong invariance model with equal thresholds was not supported, with a CFI change larger than 0.02. As suggested by Putnick and Bornstein (2016), we further tested a partial strong invariance model by releasing intercept constraints of three items from the scales of problematic social media use [35]. As indicated in Table 3, the partial strong invariance was supported, as its overall model fit was not significantly worse than the metric invariance model (ΔCFI = 0.015, ΔRMSEA = 0.003). Taken together, the results support the longitudinal measurement invariance of the constructs across time.

**Fig. 1:**
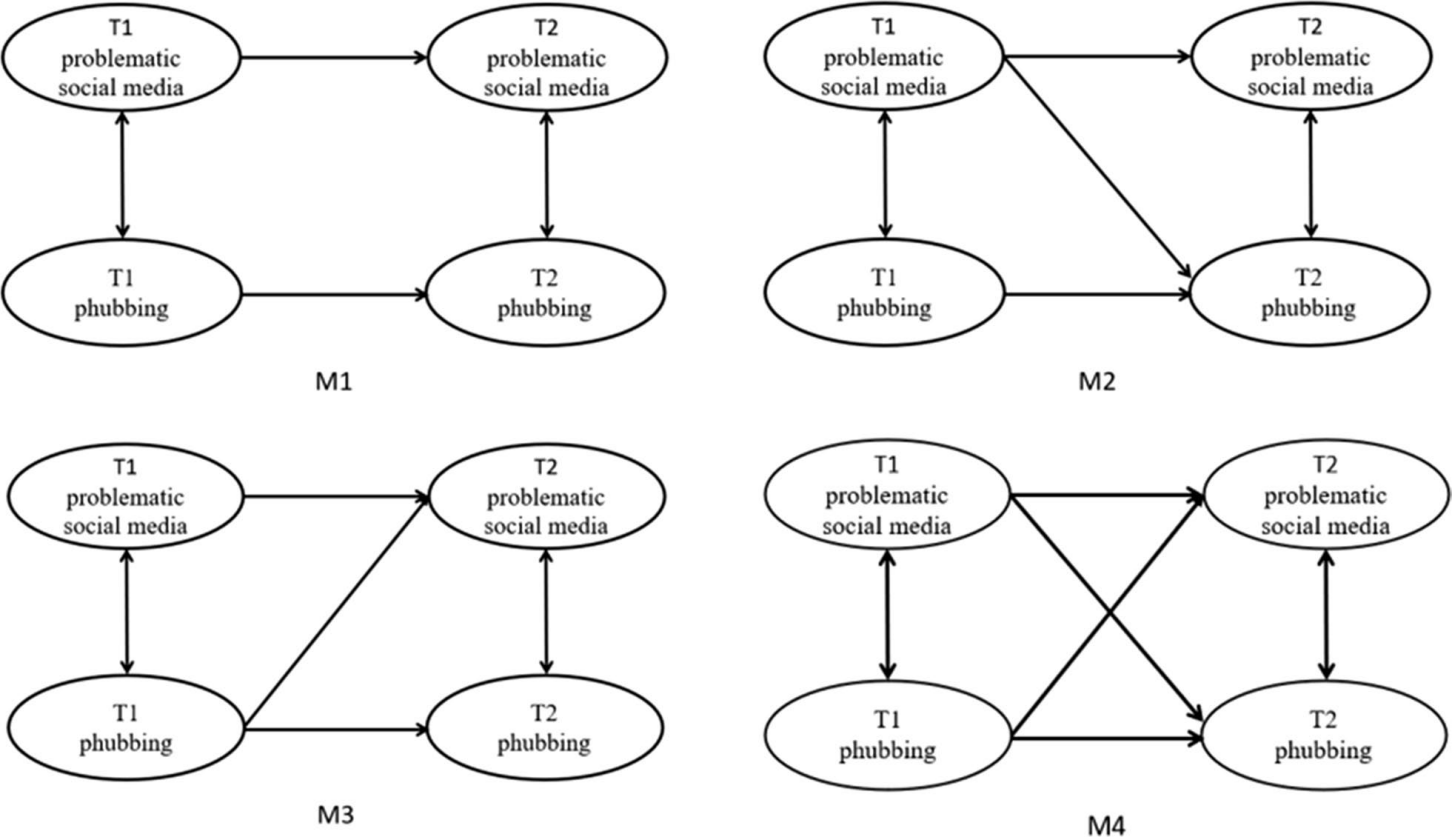
Nested models of the relationship between problematic social media use and phubbing

To explore more deeply the reciprocal prediction relationship between problematic social media use and phubbing of college students, this study adopted the design method of the cross-lagged path model and proposed four theoretical models to test the direction of the relationship between problematic social media use and phubbing (as in Fig. 1). In sum, M1 is the baseline model, assuming that early problematic social media use and phubbing can predict subsequent problematic social media use and phubbing, respectively, to test the cross-sectional stability of the two factors between the T1 and T2 time points; M2 explores the effect of T1 problematic social media use on T2 phubbing on the basis of M1; M3 explores the effect of T1 phubbing on T2 problematic social media use on the basis of M1; and M4 explores the cross-lagged relationship between problematic social media use and phubbing; i.e., it is the full model that includes all the paths described in the above mentioned models. The model with the best fitting result is the final model.

As shown in Table 4, the fit indices of all four models were acceptable. The M1 baseline model fit well, and chi-square difference tests indicated that M4 was a better fit than both M1 (Δχ^2^ = 13.427, Δdf = 2, *P* < 0.01) and M3 (Δχ^2^ = 7.887, Δdf = 1, *P* < 0.05). There was no significant difference between M4 and M2, but the fitted value of M4 was better than that of M2. Overall, M4 was the model with the best fitting results among the four models.

**Table 4:** Nested models of the relationship between problematic social media use and phubbing

Model	χ^2^	df	CFI	SRMR	RMSEA	Comparisons	Δχ^2^	Δdf	*P* value
M1	854.625	386	0.910	0.061	0.061	M4 versus M1	13.427	2	< 0.01
M2	842.695	385	0.912	0.050	0.060	M4 versus M2	1.497	1	> 0.05
M3	849.085	385	0.911	0.054	0.061	M4 versus M3	7.887	1	< 0.05
M4	841.198	384	0.912	0.049	0.060				

**Fig. 2:**
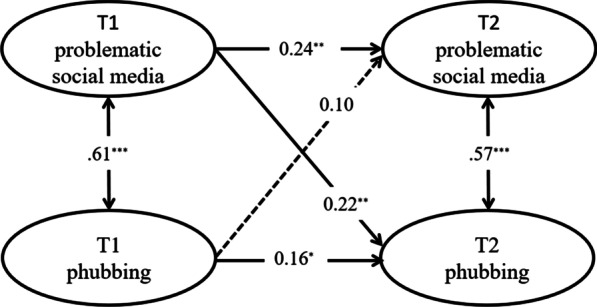
Cross-lagged relationship between problematic social media use and phubbing among college students

A cross-lagged model was constructed to analyse the reciprocal relationship between problematic social media use and phubbing among college students using Mplus 8.3 software, in which gender and age were added to the model as control variables. In this model, the same observed variable at two time points and the latent variable at the same time point were allowed to be error correlated. The model fit was examined using the great likelihood method, and the model fit well for all indicators (χ^2^ = 841.20, df = 384, CFI = 0.91, SRMR = 0.05, RMSEA = 0.06). The predictive effect of T1 problematic social media use on T2 problematic social media use was statistically significant (β = 0.24, *P* < 0.01), and T1 phubbing had a statistically significant predictive effect on T2 phubbing (β = 0.16, *P* < 0.05); furthermore, T1 problematic social media use had a statistically significant predictive effect on T2 phubbing (β = 0.22, *P* < 0.01), and T1 phubbing had no statistically significant predictive effect on T2 problematic social media (β = 0.10, *P* > 0.05). See Fig. 2.

## Discussion

Most previous studies on problematic social media use and phubbing among college students have adopted a cross-sectional design and failed to draw conclusions about the trends and reciprocal relationships between the two. This study conducted two waves of longitudinal data on Chinese college students to reveal the trends of problematic social media use and phubbing over time and the reciprocal relationship between them to gain a deeper understanding of the relationship between them.

In the descriptive statistical analysis, it was found that although the problematic social media use scores of college students increased at time point T2 compared to time point T1, the difference between the two was not statistically significant, while the phubbing score significantly decreased. This indicates that while college students' problematic social media use increased during college, their phubbing improved significantly. In high school, due to the pressure of higher education and parental control, high school students have less access to social media using smartphones, while in college, especially in the early stage of college, there is no parental and teacher control over college students' social media use; this change in control may cause a certain degree of increase in problematic social media use. However, phubbing improves during college, which may be related to the gradual establishment and development of peer relationships among college students during their school years. In college, interpersonal relationships are dominated by peer relationships, and peer relationships have an important impact on their psychological well-being [36, 37]. The most important feature of phubbing is to play with smartphones while ignoring others in social situations, while good peer relationships can increase the frequency of communication between peers and enhance the intimacy between them [38], which in turn improves this state of coldness among interpersonal relationships and thus reduces the degree of phubbing. In addition, the results of correlation analysis showed that there was a positive correlation between problematic social media use and phubbing among college students at both the T1 and T2 time points, indicating that problematic social media use and phubbing among college students have stability across time.

In the autoregressive path analysis, it was found that T1 problematic social media use significantly predicted T2 problematic social media use. This might indicate that if the problematic social media use of college students is not controlled by intervention, it will gradually deteriorate and may eventually evolve into severe psychological abnormalities and behavioural problems. Therefore, the prevention and intervention of problematic social media use among college students is essential, and the lack of timely and effective intervention may produce a slippery slope effect and increase the difficulty of its correction. In addition, T1 phubbing can significantly predict T2 phubbing, which indicates that college students' phubbing is a stable behavioural trait of individuals and is not prone to change with the influence of time and environment and other factors. Therefore, the prevention and intervention of phubbing among college students is essential, and the lack of timely and effective intervention may produce a slippery slope effect and increase the difficulty of its correction.

Cross-lagged analysis revealed that T1 problematic social media use positively predicted T2 phubbing, but T1 phubbing did not have a statistically significant predictive effect on T2 problematic social media use. First, problematic social media use among college students significantly predicted phubbing, which further deepened the results of the cross-sectional study [23, 24]. Previous causal relationships found between problematic social media use and phubbing based on cross-sectional data should be treated with caution; this study explores this issue further and more effectively. Similar studies have also shown that individuals with high levels of problematic social media use may have problems with work, academic performance, and relationships [39], and problematic mobile technology use factors are seen as effective predictors of generating phubbing. Previous research has found that social media has the function of creating social identities for users; due to the overuse of social media, people are increasingly relying on social media platforms to construct their social identities in place of identity construction in real-life scenarios [40]. The way in which people construct their social identities gradually shifts from real-life to online social media platforms, and this shift can lead to the phenomenon that people in interpersonal scenarios focus more on their smartphones and leave others out. From another perspective, problematic social media use is still one of the problematic aspects of smartphones. Numerous studies have confirmed that individuals with irrational smartphone use are more likely to snub others in social situations [22]. It is not easy for individuals with the problematic use of social media to put down their smartphones; in addition to internal factors, the design of modern electronic devices makes it easier to be alerted to new messages. Both internal brain factors and external factors of the device are powerfully attractive to smartphone users [25], and individuals are more likely to check their smartphones in social contexts while ignoring those around them. Therefore, when individuals are addicted to social media, they are equally influenced by internal and external factors and cannot resist the temptation of social media even in social situations, leading to the issue that they cannot resist touching their smartphones to check messages even in social situations; thus, they are prone to ignore the people around them due to using their smartphones, thereby destroying their social relationships in the moment. In addition, the cross-lagged analysis also found that T1 phubbing did not significantly predict T2 problematic social media use. This finding indicates that previous phubbing does not have an effect on subsequent problematic social media use; thus, a subsequent search should be conducted to find the reasons affecting problematic social media use among college students from other aspects.

There are also some shortcomings in this study. First, due to the author's resource limitation, this study selected college students from 4 colleges located within a single province as the sample using the whole group sampling method, which makes the sample of this study slightly less representative. As the phenomenon of phubbing is already a geographically and gender-neutral phenomenon, other samples should be added in the future to verify whether the influence relationship also exists in different age groups and within different cultural backgrounds. Second, the study used a self-report method to collect data, which is a highly subjective measurement; thus, some biases of individuals may affect the results. In the future, we may consider multiple methods to collect data for the study together, such as using a paired survey to study phubbing calling among friends. Finally, this study conducted only one follow-up and did not examine the developmental trajectory of each variable across the 20-month follow-up period. In the future, we need to increase the number of years and times of follow-up in studies to explore the relationship between problematic social media use and phubbing among college students more deeply.

## Conclusion

This study found that (a) baseline levels of phubbing among college students significantly and positively predicted developmental levels 20 months later, whereas baseline levels of problematic social media use did not significantly predict developmental levels 20 months later. Furthermore, (b) problematic social media use significantly and positively predicted the level of phubbing 20 months later.

## Data Availability

The data that support the findings of this study are available on request from the corresponding author WANG. The data are not publicly available due to them containing information that could compromise research participant privacy.

## Abbreviations

*P*: *P* Value
SRMR: Standardized root mean square residual
RMSEA: Root mean square error of approximation
CFl: Comparative fit index
